# Bronchial collapse and bronchial stenting in 9 dogs

**DOI:** 10.1111/jvim.16859

**Published:** 2023-09-11

**Authors:** Darren Kelly, Florence Juvet, Valerie Lamb, Andrew Holdsworth

**Affiliations:** ^1^ Southern Counties Veterinary Specialists Ringwood United Kingdom; ^2^ Davies Veterinary Specialists Shillington United Kingdom

**Keywords:** airway collapse, bronchomalacia, mainstem bronchus, tracheal collapse

## Abstract

**Background:**

Principal and lobar bronchial collapse is increasingly recognized as an isolated entity.

**Objective:**

Retrospectively describe the procedure and outcomes of dogs undergoing bronchial stenting at a single referral hospital.

**Animals:**

Nine client‐owned dogs with variable degrees of collapse of the left principal bronchus (LPB), lobar bronchus 1 (LB1), and lobar bronchus 2 (LB2), and with clinically relevant signs of respiratory dysfunction.

**Methods:**

Data were collected from patient records. All dogs underwent stenting of the LPB and LB2. Anatomic and functional impairment grades were assigned to each case before and 4 weeks after stenting. Data regarding response to stenting and complications were evaluated.

**Results:**

Bronchial stenting was considered successful in all cases, with all dogs experiencing improved quality of life (QOL), and decreased functional impairment grade at 4 weeks post‐stenting. Follow‐up of >6 months was available for 6 dogs and of these, 5 were alive at 12 months, 3 were alive at 18 months, and 1 was alive at 24 months. Stent‐related complications occurred in 4 dogs, and were resolvable in 3. Two dogs developed pneumothorax, 1 developed recurrent pneumonia, and 1 developed new‐onset coughing. All dogs had mild and manageable coughing post‐stenting.

**Conclusions and Clinical Importance:**

Stenting of the LBP and LB2 might be an effective option for dogs with advanced collapse of these bronchi and associated signs. Although all included dogs had resolution or improvement of clinical signs considered life‐threatening or as affecting QOL, ongoing coughing is expected. Patient selection appears important with regard to achieving successful outcomes.

**Abbreviations**
BALF: bronchoalveolar lavage fluid
CHF: congestive heart failure
CT: computed tomography
FIG: functional impairment grade
IAD: inflammatory airway disease
LA: left atrial
LB1: lobar bronchus 1
LB2: lobar bronchus 2
LPB: left principal bronchus
MMVD: myxomatous mitral valve disease
QOL: quality‐of‐life
RPB: right principal bronchus

## 1 INTRODUCTION

Mainstem bronchial collapse, lobar bronchial collapse, and bronchomalacia were reported as isolated clinical entities in 2010.^1^ Publications on this topic have increased but remain scarce, particularly considering bronchial collapse appears to be more common than tracheal collapse in dogs investigated for respiratory disease with bronchoscopy.^1^, ^2^ There is discrepancy regarding the terminology used in much of the previously published literature on the topic, with previous studies using the term bronchomalacia when discussing collapse of any portion of the airway distal to the trachea.^1^, ^2^, ^3^, ^4^, ^5^ A recent article reviewed this topic, and proposed definitions for type of collapse, area affected, anatomic grading, and functional impairment grading.^6^ It proposed that bronchomalacia in dogs be defined as regional to diffuse dynamic airway collapse of segmental and subsegmental bronchi with associated clinical signs caused by airflow limitation.^6^ It suggested that collapse of larger airways, proximal to segmental bronchi, be defined by location, type of collapse, and grade. In accordance with these definitions, collapse of a mainstem bronchus is defined as static or dynamic collapse of a left or right principal bronchus (RPB), and collapse of a lobar bronchus is defined as static or dynamic collapse of a lobar bronchus along with identification of the supplied lobe.^6^

The etiology of bronchial collapse and bronchomalacia is unknown, but associations have been described, and hypotheses regarding etiopathogenesis proposed. Lower airway collapse is commonly identified with tracheal collapse, and it has been speculated that the cartilaginous defect affecting dogs with tracheal collapse might extend distally to affect the bronchi in some cases.^1^, ^7^ Histologic assessment of bronchial cartilage has been reported in very few dogs with lower airway collapse, and the presence of cartilaginous abnormalities appears inconsistent.^3^, ^6^, ^8^, ^9^ A causal relationship between left atrial (LA) enlargement caused by myxomatous mitral valve disease (MMVD), and subsequent compression of the left mainstem bronchus, has been suggested in some published literature and textbooks.^10^, ^11^ Evidence to support this hypothesis is lacking. One study found no association between LA size and airway collapse in dogs with MMVD presenting for cough,^4^ whereas another found an association (without proving causation) between increasing LA size and decreasing bronchial lumen diameters on computed tomography (CT).^12^ Multiple case series have described dogs with collapse of principal or lobar bronchi or both in the absence of cardiomegaly.^1^, ^2^, ^3^, ^5^ Currently, no clear association has been established between airway infection and bronchial collapse. Two previous studies identified infection relatively commonly (56%‐62% of samples),^2^, ^3^ whereas 2 others found infection to be uncommon (6%‐10% of samples).^1^, ^4^ Regarding inflammatory airway disease (IAD) and chronic bronchitis, existing literature documents inflammatory change in the majority of dogs with lower airway collapse, ranging from 85% to 100% of affected dogs.^1^, ^2^, ^4^ Although IAD and airway collapse are commonly documented together, 1 study with a design allowing determination of association, did not detect an association between them.^1^ Obesity has detrimental effects on respiratory function, limiting airflow during the expiratory phase of respiration, and increasing bronchial reactivity in dogs.^13^, ^14^ A causal relationship between obesity and airway collapse has not been identified, and 1 study found no significant difference in body condition scores between patients with airway collapse and a control group.^1^ Despite this finding, and considering the negative effects on airway function, it is worth noting that approximately 50% of dogs with lower airway collapse are overweight or obese.^2^, ^3^ Static collapse of the mainstem or lobar bronchi or both, with or without associated clinical signs, is common in brachycephalic dogs.^15^, ^16^ It seems feasible that bronchial abnormalities in brachycephalic dogs might have a different etiopathogenesis as compared to non‐brachycephalic breeds, and might be related to their conformation.^6^, ^15^, ^17^

Clinical signs reported in dogs with bronchial collapse and bronchomalacia include coughing,^1^, ^2^, ^3^, ^4^, ^5^, ^6^ persistent dyspnea,^5^, ^6^ episodic dyspnea,^3^, ^5^ cyanosis,^3^, ^6^ abnormal respiratory noises,^6^ syncope,^6^ and exercise intolerance.^3^ An association between the development of pulmonary hypertension and bronchomalacia also has been suggested.^6^, ^18^, ^19^ In recent years, we have increasingly identified dogs with lower airway collapse presenting with some combination of the aforementioned clinical signs. Herein, we report the findings and outcomes of such dogs that have undergone bronchial stenting at a single referral hospital.

## 2 MATERIALS AND METHODS

We have been offering bronchial stenting since January 2020. All dogs in which bronchial stenting was performed at our hospital until the time of manuscript preparation (January 2023) were identified for inclusion. Dogs that had tracheal stenting performed, either before or at the same time as bronchial stenting, were excluded. The standard hospital admission form used for all dogs admitted to hospital and signed by all owners included provision of consent for retention and use of all patient data for future teaching or scientific publication purposes.

Data collected retrospectively from medical records included signalment, reason for presentation, clinical signs, cardiac status, diagnostic imaging findings, bronchoscopy findings, bronchoalveolar lavage fluid (BALF) results, medications prescribed, follow‐up information, and information regarding stent or procedure‐related complications.

The diagnosis of bronchial collapse was based on bronchoscopy in all cases. Bronchoscopy recordings were reviewed by the primary author (DK) at the time of manuscript preparation, for the purpose of confirming the type, anatomic location, and grade of collapse. The anatomic definitions and grading scheme (grade I‐III) proposed previously were used.^6^ Herein, the left mainstem (principal) bronchus is designated left principal bronchus (LPB), the left cranial lobar bronchus is designated LB1, and the left caudal lobar bronchus is designated LB2. A modification of the functional impairment grading scheme previously proposed^6^ was used (Table 1), with the addition of a grade to distinguish those dogs that had been hospitalized for emergency treatment because of perceived life‐threatening clinical signs on at least 1 occasion. Each dog was retrospectively assigned a functional impairment grade (FIG).

**TABLE 1:** Functional impairment grading scheme.

Grade	Description
0	Lacking clinical signs of respiratory disease
1	Spontaneous, intermittent less severe clinical signs (ie, cough, excessive panting, tachypnoea) interspersed by long periods of normalcy
2	Clinical signs inducible with excitement or exertion
3	Clinical signs triggered by routine daily activities
4	Persistent clinical signs at rest
5	Clinical signs resulting in at least 1 episode of hospitalization for treatment

*Note*: Grading scheme modified from Reinero and Masseau (2021)^6^ with the addition of a grade to differentiate those patients which required hospitalization on 1 or more occasions because of perceived life‐threatening clinical signs.

Echocardiography was performed by a cardiology diplomate, and reported in accordance with the 2019 consensus statement regarding the diagnosis of MMVD.^20^ A diagnosis of concurrent IAD was made if BALF analysis showed that >12% of nucleated cells were neutrophils.^21^, ^22^

**FIGURE 1:**
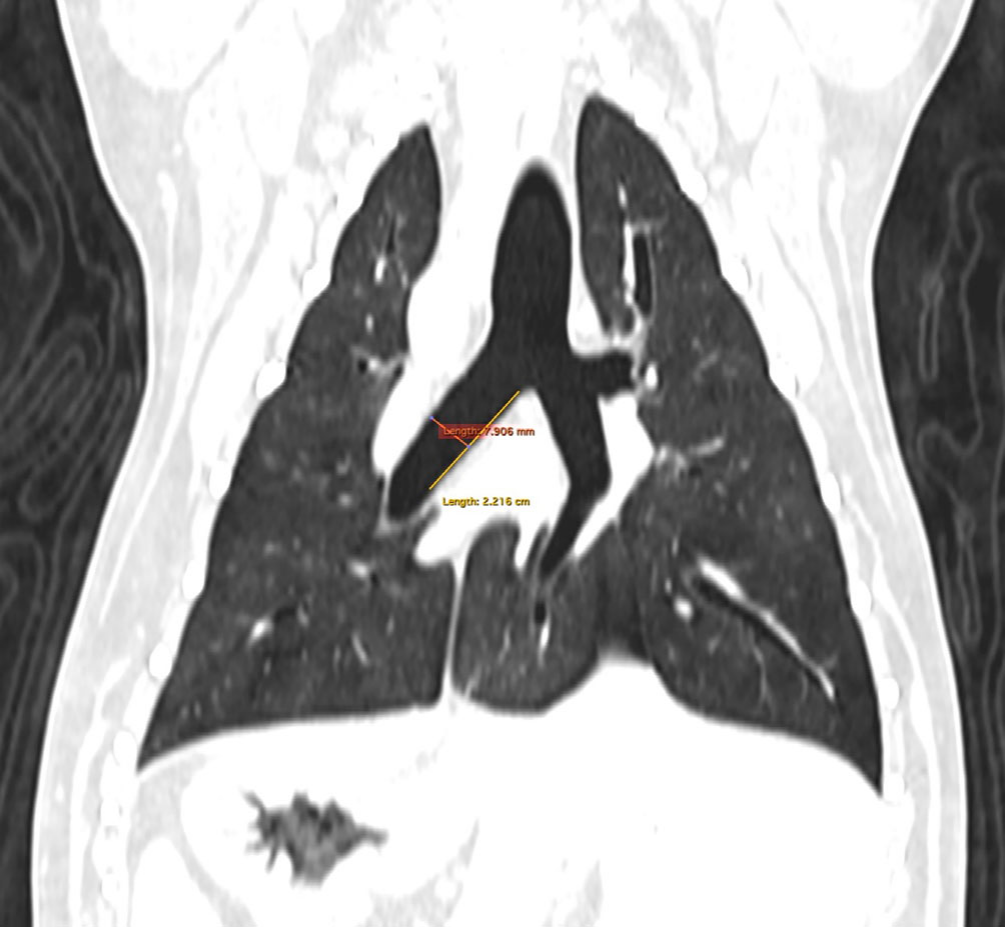
Dorsal plane computed tomography image taken from a measurement scan with the patient ventilated to a pressure of 20 cm H_2_O. The yellow line spans the length of the left principal bronchus and lobar bronchus 2, and measures the maximal length of the area to be stented. The red line measures the maximal diameter of the bronchus at its widest part.

Computed tomography scans were reviewed by 1 of the authors (AH). Computed tomography of the chest was performed in all cases, using a 64‐slice helical scanner (Somatom; Siemens, Germany) with standard settings of 160 mAs, 130 kV, pitch factor 0.6, collimation width 1.2 mm, and scan time of 10‐12 seconds. Two scans were performed in each dog: a diagnostic scan and a measurement scan. Images acquired during the diagnostic scan were interpreted and reported with the intention of identifying abnormalities (aside from airway collapse) that could explain the patient's signs. For the diagnostic scan, the patient was hyperventilated to induce apnea. A ventilator was used for all cases, with ventilation to a normal positive end‐expiratory pressure to prevent atelectasis. For the measurement scan, used to measure the maximal diameter and length of the section of airway to be stented, the patient was hyperventilated to induce apnea. The airway was ventilated to a pressure of 20 cm H_2_O or until the trachea, mainstem, and lobar bronchi appear maximally inflated. Figure 1 shows the target stent position for all cases and a suitable imaging plane for measurements.

In all cases, the entirety of the LPB and LB2 was stented. A stent with a fully expanded diameter of 1‐2 mm more than the maximal diameter of the area to be stented was chosen. A shortening chart provided by the stent manufacturer was used when selecting a stent size for placement, to ensure optimal stent length after deployment and expansion to the maximal diameter of the section of airway to be stented. Self‐expanding, uncovered, nitinol stents, marketed for use in the bronchus of veterinary patients, were used (Vet Stent‐Bronchus, Infiniti Medical, Menlo Park, California).

**FIGURE 2:**
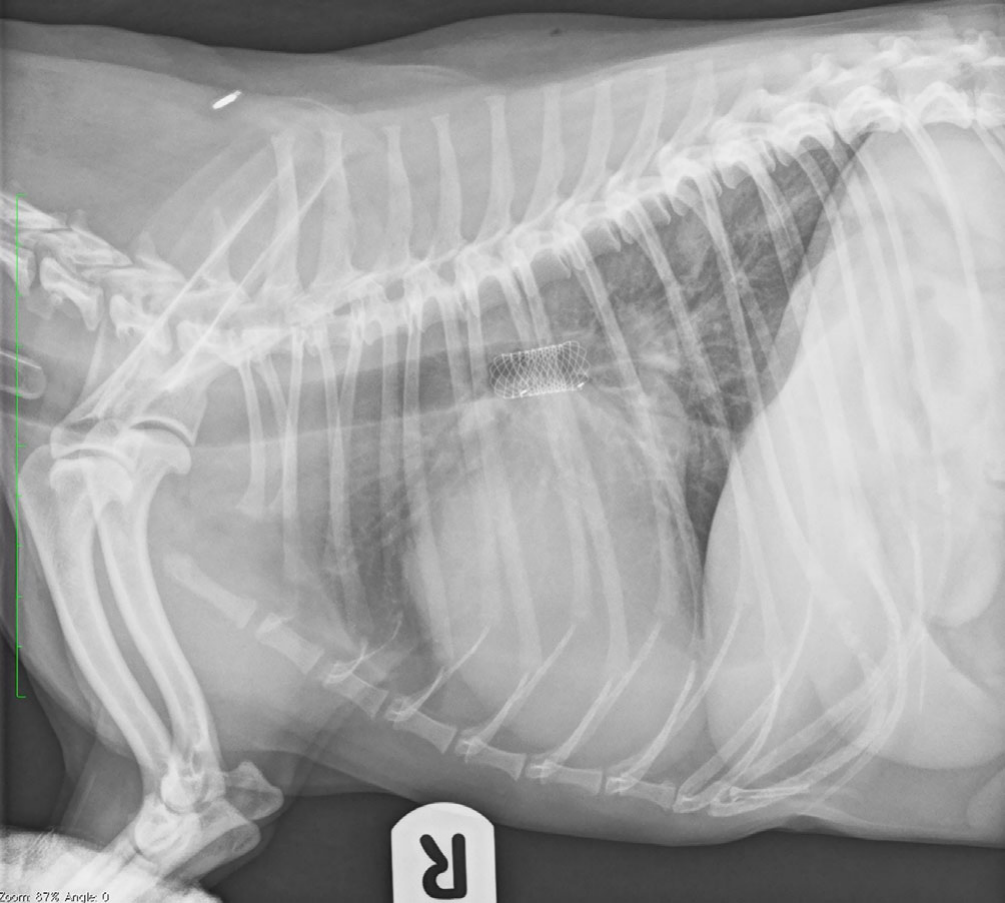
Right lateral radiograph demonstrating the position of a bronchial stent.

**FIGURE 3:**
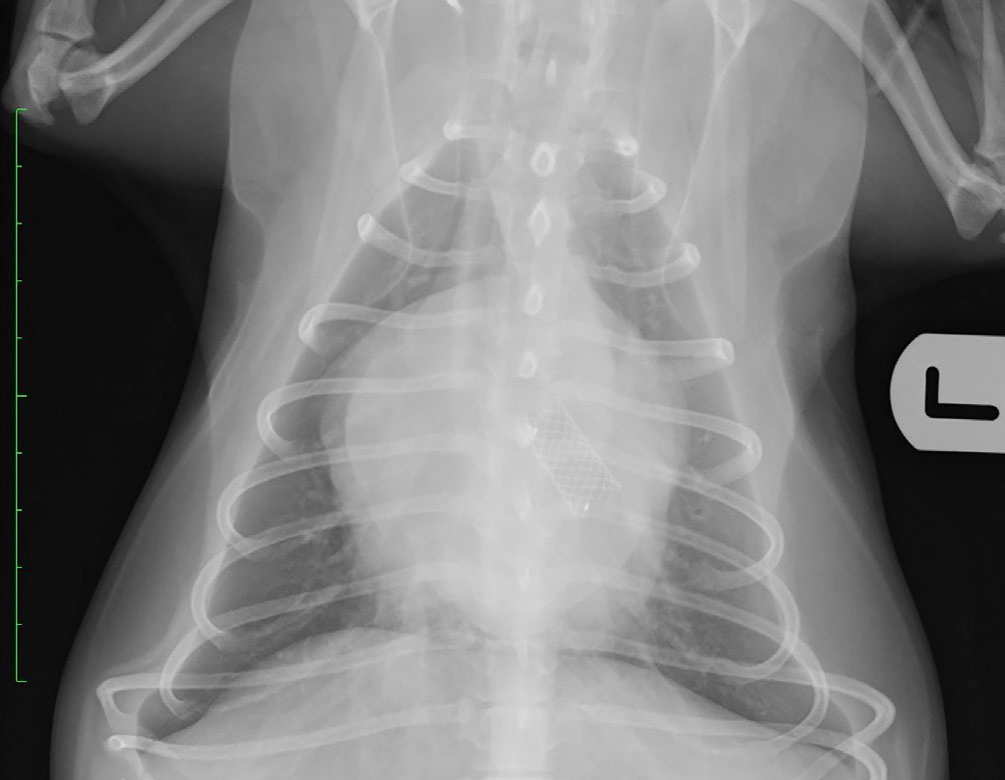
Dorsoventral radiograph demonstrating the position of a bronchial stent.

All stents were placed with the patient anesthetized and in sternal recumbency. Bronchoscopy and dorsoventral fluoroscopic images were used during stent placement. Briefly, the steps taken during stenting were as follows: Step 1: flexible bronchoscopy was performed, either with the patient extubated or through an endotracheal tube. Step 2: the bronchoscope was passed to the level of the carina or most proximal aspect of the LPB, and a piece of tape was used to mark the bronchoscope at this level. Step 3: the bronchoscope was passed to the most distal aspect of LB2 (to the point where it starts to divide), and a piece of tape was used to mark the bronchoscope at this level. Step 4: the distance between the 2 pieces of tape placed on the bronchoscope was measured to ensure the length of stent chosen based on the CT measurement was equivalent to the length of section to be stented based on this method of measurement. Step 5: the bronchoscope was withdrawn to the level of the carina or most proximal aspect of the LPB and, using fluoroscopy to confirm correct positioning, a 23 g needle was placed SC on the dorsum to mark the point of the distal end of the bronchoscope on the fluoroscopy images. Step 6: the bronchoscope was passed back to the most distal aspect of LB2 and, using fluoroscopy to confirm correct positioning, a 23 g needle was placed SC on the dorsum to mark the point of the distal end of the bronchoscope on the fluoroscopy images. It is important that the patient's skin is not moved after this point, until completion of the procedure. Step 7: a 0.025 inch hydrophilic guidewire (Weasel Wire, Infiniti Medical, Menlo Park, California) was passed through the working channel of the bronchoscope and into 1 of the divisions of LB2. Step 8: the bronchoscope was withdrawn over the guidewire and completely removed, with fluoroscopy images obtained intermittently to ensure the position of the guidewire remained unchanged. Step 9: the constrained stent on the deployment device was passed over the guidewire and into the LPB and LB2. Step 10: the stent was deployed under continuous fluoroscopic guidance, with the previously placed SC needles marking the aimed targets for the distal and proximal ends of the stent. Step 11: the guidewire and stent deployment device were removed. Step 12: before being recovered from anesthesia, bronchoscopy was repeated to evaluate positioning of the stent, and thoracic radiography was performed immediately post‐stenting in the majority of cases. Fluoroscopy clips (Video S1) are available in the supporting information showing deployment of bronchial stents in 2 dogs, and Figures 2 and 3 show thoracic radiographs taken immediately after bronchial stenting.

Bronchial stenting was considered successful if the patient's FIG 4 weeks post‐stenting was decreased to ≤ grade 3. Any unwanted or unexpected occurrence affecting the patient during or in the 24 hours after stenting, new onset of any clinical sign potentially attributable to stenting, or progression of any previously reported clinical sign were considered complications.

## 3 RESULTS

### 3.1 Cases

Between January 2020 and January 2023, we placed bronchial stents in 13 dogs. Four were excluded because they also had tracheal stents placed. Nine dogs were included in this case series. The signalments, clinical signs, results of diagnostic testing, and case outcomes are summarized in the Supporting Information. All dogs had been prescribed various combinations of medications before stenting, including antimicrobials, corticosteroids, non‐steroidal anti‐inflammatory drugs, antitussives, diuretics, and pimobendan.

### 3.2 Clinical signs

Coughing was reported in 8/9 dogs. Decreased exercise tolerance was reported in 7/9 dogs. Spontaneously resolving episodic dyspnea (owner‐reported difficulty breathing) was reported in 5/9 dogs, with owners frequently describing such episodes as their dog apparently choking. Dyspnea (without evidence of concurrent congestive heart failure [CHF]) at presentation and immediately before stenting, or at least 1 previous episode of dyspnea (without evidence of concurrent CHF) requiring veterinary attention, was present in 4/9 dogs. A recent onset of stertorous breathing or snoring was reported in 3/9 dogs. Syncopal episodes were reported in 2/9 dogs.

### 3.3 Cardiac status at the time of stenting

Based on the absence of a heart murmur, and no evidence of cardiomegaly on imaging, 4/9 dogs were assessed as not having clinically relevant structural heart disease. One dog was diagnosed with stage B1 MMVD, 2 with stage B2 MMVD, and 2 with stage C MMVD. No patient had evidence of CHF on thoracic radiographs or CT immediately before stenting. Both dogs with stage C MMVD met the criteria for a clinical diagnosis of pulmonary hypertension, having an intermediate or high probability of pulmonary hypertension and a tricuspid regurgitant velocity >3.4 m/s.^18^

### 3.4 Bronchoscopy findings

Bronchoscopy was performed in all dogs. The LPB, LB1, and LB2 were collapsed to variable degrees in all cases. Of the 9 dogs, LB2 had grade III collapse in 9, the LPB had grade II collapse in 9, and LB1 had grade III collapse in 7, grade II collapse in 1, and grade I collapse in 1. Grade I bronchomalacia was identified in 2 cases, and grade I tracheal collapse was identified in 2 cases.

### 3.5 Concurrent IAD

Of the 9 dogs, BALF analysis results were available for 5, with 4/5 showing variable degrees of neutrophilic inflammation.

### 3.6 Outcomes

Stenting of the LPB and LB2 was performed by 1 of the authors (DK or FJ) in all 9 cases. Stenting was considered successful in 9/9 dogs, with all having a decrease in their FIG by at least 2 grades at 4 weeks after stenting. Of the 9 dogs, all were alive at 1 month. For 3 cases, <6 months follow‐up was available at the time of manuscript preparation. Of the other 6 dogs, 5/6 were alive at 6 months and 12 months, 3/6 were alive at 18 months, and 1/6 was alive at 24 months. In all of the dogs presenting with dyspnea, or in which episodic dyspnea was reported, resolution of these signs occurred. Of the dogs that were coughing before stenting, the cough was reported to be substantially improved in 7/8 cases, and stable in 1/8 cases. All dogs were reported to have occasional coughing after stenting, but all owners considered this manageable and not substantially affecting the dog's quality of life (QOL). The 3 dogs that were reported to have snoring or stertorous breathing had resolution of these signs. Exercise tolerance was considered improved for the 7 dogs in which this clinical sign was reported before stenting.

### 3.7 Complications

Complications occurred in 4/9 dogs. No complication resulted in death of the patient, and complications were resolvable in 3/4 cases. Quality of life was considered substantially affected because of a complication in 1/9 dogs, but the patient's QOL improved substantially after resolution of the complication. Two dogs developed pneumothorax either during or immediately after the stenting procedure. In 1 case, pneumothorax was noticed on fluoroscopy images during the stenting procedure. In another case, pneumothorax was documented on thoracic radiographs that were acquired because the patient became dyspneic on recovery. Thoracocentesis resulted in complete resolution of pneumothorax in both patients. The occurrence of pneumothorax did not result in prolongation of hospitalization in either patient, and neither required placement of an indwelling thoracostomy tube. One dog, in which coughing was not reported before stenting, developed coughing after stenting. The owner reported 1‐2 cough episodes per day, considered the cough very mild and as not affecting the dog's QOL, and did not require any specific medication or intervention. The first dog in which bronchial stenting was performed at our hospital developed recurrent bacterial pneumonia affecting the right lung. In this case, 2 bronchial stents were placed. The first stent placed in this patient was considered too proximally positioned, with the proximal end of the stent within the distal trachea, and distal end of the stent at the junction between the LPB and LB2. This inadvertent positioning resulted in the stent completely crossing the entrance of the RPB. Initial attempts to remove this malpositioned stent bronchoscopically, immediately after deployment, were unsuccessful and the decision was made to place a second stent through and overlapping the first, and this stent was considered appropriately positioned. The inappropriately positioned stent was removed after 2 separate attempts.

## 4 DISCUSSION

Since 2000, many publications have described the use of intraluminal stents to manage tracheal collapse in dogs. The first description of the placement of stents into the bronchi of healthy dogs was an experimental study published in the human medical literature in 1986.^23^ The first description of bronchial stenting in the veterinary medical literature was over 25 years ago, and was again an experimental study using healthy dogs.^24^ Although the topic of bronchial stenting is discussed in a major veterinary interventional radiology textbook,^25^ at the time of manuscript preparation, there was only a single case report describing the clinical use of a bronchial stent in a dog.^26^

Herein, we report the findings, technique, and outcomes of 9 dogs that underwent bronchial stenting. All dogs had variable severities of collapse of the LPB, LB1, and LBP, in the absence of concurrent clinically relevant tracheal collapse or diffuse bronchomalacia. All dogs had high FIGs, with 8 assigned a FIG of 4 or 5 before stenting, and with all owners considering their dogs' QOL to be substantially affected. Although 1 included dog had a FIG of 3 before stenting, this dog had severe exercise intolerance and the owner considered the dog's QOL to be substantially affected. Bronchial stenting was considered successful in all 9 cases, with all dogs assigned a FIG of ≤3 at 1 month post‐stenting. We believe this apparent high success rate is related to appropriate case selection. Dogs with high anatomic and functional impairment grades, and those without concurrent substantial tracheal collapse or bronchomalacia, appear to be potentially suitable candidates for bronchial stenting.

Tracheal stenting is an effective management option for dogs with advanced tracheal collapse syndrome.^27^, ^28^, ^29^, ^30^, ^31^ We have placed bronchial stents in dogs with tracheal stents, but these cases were not included for several reasons. Because none of the dogs included in our report had concurrent clinically relevant tracheal collapse, it seems likely that the etiology and natural disease progression of their airway collapse differ from those with substantial tracheal collapse and concurrent collapse of the lower airways, and the dogs included seem to have a different disease process than those that have undergone both tracheal and bronchial stenting. We are reluctant to make comparisons between the outcomes of cases included in this report with those reported previously for tracheal stenting, as the appropriately termed tracheal collapse syndrome itself appears to have different etiopathogeneses.^31^, ^32^ Considering the relatively high complication rate associated with tracheal stenting,^27^, ^28^, ^30^, ^31^, ^33^ we felt that including dogs that also had tracheal stents in place might make it difficult to evaluate and attribute any complications and associated clinical signs to bronchial stenting.

Complications occurred in 4/9 cases. Two dogs developed pneumothorax, which resolved after thoracocentesis. In both cases, we suspect the pneumothorax was induced iatrogenically by the guidewire. One dog had clinically relevant complications because of a malpositioned stent. The owner initially reported good progress, but after 10 months the dog developed severe coughing and lethargy, and investigations confirmed bacterial pneumonia affecting the right lung, with purulent secretions visibly trapped in the RPB because of the malpositioned stent. Because of recurrent episodes of pneumonia affecting the right lung and associated poor QOL, the decision was made after 15 months to repeat the attempt to remove the malpositioned stent and to euthanize the dog if this attempt was unsuccessful. Attempts to remove the malpositioned stent using a grabbing device passed through the working channel of the bronchoscope were repeatedly unsuccessful, but the stent then was successfully retrieved by grasping it with an alligator forceps and applying traction. The second appropriately positioned stent remained in place and was unchanged after removal of the malpositioned stent. The dog recovered uneventfully from the stent retrieval procedure, and was alive with good QOL, no further episodes of pneumonia, and ongoing resolution of the initial clinical signs, at the time of manuscript preparation (31 months after stenting). Given the difficulty associated with removal of this malpositioned stent, we recommend accuracy with regard to measuring and marking the target area for stent deployment. As our experience has increased, we have noticed that the stents tend to move slightly proximally immediately after they are deployed. Consequently, we now start to deploy bronchial stents 1‐2 mm distal to the marker denoting the distal end of LB2 and pause deployment during expiration, and doing so thus far has resulted in ideal placement in all subsequent cases. One dog that was not coughing before stenting, developed a cough after stenting. The owner described this as 1 or 2 episodes of coughing per day, and felt it did not affect the dog's QOL. No medications were prescribed, and the owners did not mention it to the local veterinarian during a routine health evaluation approximately 6 months post‐stenting.

Stent fracture is 1 of the most commonly reported complications of tracheal stenting, being reported in up to 42% of cases.^28^ Recent publications report lower rates of stent fracture, with 19%, 22%, and 25% of cases affected.^30^, ^31^, ^33^ The lower rates of stent fracture in recent literature likely reflect increased experience, better understanding of tracheal collapse syndrome and stenting, and progress with regard to stent engineering. We have not documented or suspected fracture of a bronchial stent in any dog. Although follow‐up has been inconsistent (ie, we cannot exclude the possibility that stent fracture might have occurred in some of our patients), we consider this outcome unlikely based on clinical progression. Obstructive tissue ingrowth is another potential complication of tracheal stenting in dogs, occurring in 17%‐19% of cases.^31^, ^33^ With recognition of inconsistent follow‐up, we have not documented or suspected obstructive tissue ingrowth in any included cases, but we have documented obstructive tissue ingrowth affecting a bronchial stent in 1 dog with a concurrent tracheal stent. We chose to discuss these 2 potential complications, considering they are the most common major complications associated with tracheal stenting. We would, however, advise caution when comparing bronchial and tracheal stenting, because disease progression, airway dynamics, and interactions between biomechanics and stent are likely to differ substantially with these conditions.

Ongoing coughing was reported in all patients, but was considered mild and manageable in all. Coughing was reported as a clinical sign in 8/9 cases, with the owners of 3 of these dogs considering the cough severe and affecting their dog's QOL despite medical treatment. Although an improvement with regard to coughing was reported in 7/8 cases, warning owners that coughing is not expected to resolve is prudent in order to manage expectations. The exact cause of the ongoing coughing is unknown, but persistent (eg, LB1) or progressive airway collapse, concurrent airway inflammation, airway irritation because of the stent, or any combination of these, are considered most likely. Of the 5 dogs previously diagnosed with heart disease, none had evidence of CHF at the time of stenting. We agree with the opinions expressed in a 2018 review article discussing the evidence for (or apparent lack of) a causal relationship between MMVD and coughing. It seems likely that coughing in dogs with MMVD is, for the most part, related to the concurrent presence of ≥1 primary respiratory diseases, such as chronic bronchitis or airway collapse.^34^ A previous study showed no association between CHF and coughing in dogs, but found an association between LA enlargement and coughing.^35^ The veterinary medical literature contains conflicting reports regarding whether or not an association exists between LA enlargement and lower airway collapse, with 1 study finding an association, and another not finding an association.^4^, ^12^ Currently, no evidence exists that LA enlargement causes airway collapse in dogs. Although any association between LA enlargement and coughing might merely reflect a particular population simultaneously being predisposed to MMVD and primary airway disease, it also seems feasible that LA enlargement could contribute to coughing in some dogs with pre‐existing and unrelated bronchial pathology. In this small case series, 4/5 dogs for which BALF was available had neutrophilic inflammation, which is similar to the previously reported high prevalence of inflammatory changes in dogs with lower airway collapse.^1^, ^2^, ^4^ We have used the term IAD, because included cases with inflammation on BALF analysis do not meet the criteria frequently used to diagnose chronic bronchitis, considering another potential explanation for the cough was present.^36^ Interestingly, the only dog that had normal BALF cytology results was also the only dog not reported to be coughing before stenting. Although it is impossible to draw any conclusions from this observation, it is worth noting that the cough immediately post‐stenting was reported to be extremely mild in this case, and was not reported at all during a subsequent health evaluation with the referring veterinarian. Although it has been suggested in humans that the trauma of coughing itself can trigger airway inflammation,^37^ we postulate that ongoing coughing in these dogs could be caused by concurrent IAD and airway collapse, without a causative relationship necessarily existing between the 2.

A diagnosis of lower airway collapse typically is based on bronchoscopy.^1^, ^2^, ^3^, ^4^, ^6^ Computed tomography frequently is recommended in dogs, particularly with the addition of paired inspiratory and expiratory breath‐holding scans, to assist with diagnosis, and definition of location and grade.^6^, ^38^, ^39^ The use of CT to assist with diagnosing dynamic airway collapse in humans appears to be increasing.^40^, ^41^ One recent study found limited agreement between tracheobronchoscopy metrics and paired inspiratory and expiratory CT metrics when evaluating airway collapse in dogs, with weighted kappa coefficient values falling in the poor to slight agreement or fair agreement ranges for all mainstem and lobar bronchi, and only moderate agreement for bronchomalacia. A kappa coefficient value consistent with substantial agreement was not found for any location, and the authors concluded that although inspiratory and expiratory CT scans can detect airway collapse with limited agreement with tracheobronchoscopy, additional studies correlating the severity of airway collapse with FIGs are needed.^39^ A previous study assessing collapsibility of the mainstem and lobar bronchi in clinically normal dogs using inspiratory and expiratory CT scans found the mean collapsibility of all bronchi to be 38.20 ± 15.17%, with potential collapsibility of >50% seen for both segmental divisions of LB1, LB2, and RB2.^42^ Another study also found that bronchial diameters can change in healthy dogs depending on the phase of respiration, with collapsibility values similar to those of the aforementioned study for lobar bronchi. This study documented significantly higher collapsibility values during forced expiration, with values up to 70% and 74% documented in some dogs for LB1 and LB2, respectively.^43^ Inspiratory breath‐holding and positive pressure CT scans were performed in all included cases as part of the investigation of the clinical signs, and for airway measurement to assist with stent size selection. We currently do not perform forced expiration CT scans as part of assessment and, based on the above, do not currently use inspiratory and expiratory CT scans for the anatomic diagnosis or grading of mainstem or lobar bronchial collapse. Although we recognize that inspiratory and expiratory CT scans might play a role in diagnosing bronchomalacia in the scenario in which the more distal airways cannot be reached and assessed by bronchoscopy, we feel additional studies evaluating the sensitivity, specificity, and positive and negative predictive values of this test are required.

The main limitation of our study was its retrospective design. This feature is particularly pertinent with regard to follow‐up. Because of the detail of the clinical notes available for all patients before stenting, we feel an accurate FIG was assigned to each case for before stenting. Unfortunately, follow‐up clinical notes frequently were not as detailed, and often only contained information regarding the presence or absence of clinical signs, along with a subjective comparison with findings before stenting. The absence of more detailed follow‐up data was particularly important when evaluating coughing post‐stenting, and assigning a FIG at 4 weeks. For example, follow‐up notes might contain information such as “coughing intermittently, but much improved compared to before stenting.” This type of language made it difficult in some cases to decide whether a FIG of grade 2 or grade 3 should be assigned at 4 weeks. In cases in which follow‐up information was not detailed enough to reliably distinguish between FIGs, we assigned a higher FIG rather than risk overestimating the response to stenting. Follow‐up was not standardized, and the type and timing of follow‐up varied among cases. Follow‐up diagnostic imaging or bronchoscopy or both was not performed in the majority of cases, primarily because the owners were relatively satisfied with their dogs' progress. Regardless, having routine and standardized follow‐up might have facilitated the detection of any potentially mild complications, or more subtle changes with regard to patient progress.

In conclusion, stenting of the LPB and LB2 might be a suitable management option for dogs with clinically relevant collapse of these bronchi, in the presence of high anatomic and functional impairment grades, and where medical management and ensuring an optimal body condition score has failed to control the clinical signs. Prospective studies utilizing the recently proposed definitions and with standardized follow‐up are warranted to further investigate the role of stenting in dogs with mainstem and lobar bronchial collapse.

## CONFLICT OF INTEREST DECLARATION

Authors declare no conflict of interest.

## OFF‐LABEL ANTIMICROBIAL DECLARATION

Authors declare no off‐label use of antimicrobials.

## INSTITUTIONAL ANIMAL CARE AND USE COMMITTEE (IACUC) OR OTHER APPROVAL DECLARATION

Authors declare no IACUC or other approval was needed.

## HUMAN ETHICS APPROVAL DECLARATION

Authors declare human ethics approval was not needed for this study.

## Supporting information


**Data S1:** Supporting Information.

**Video S1:** MP4 file containing 2 fluoroscopy clips showing bronchial stent deployment in 2 dogs.
